# Supplementary material for: Simultaneous Infection With Porcine Reproductive and Respiratory Syndrome and Influenza Viruses Abrogates Clinical Protection Induced by Live Attenuated Porcine Reproductive and Respiratory Syndrome Vaccination
Source: Front Immunol. 2021 Nov 11;12:758368. doi: 10.3389/fimmu.2021.758368 (PMC8632230; doi:10.3389/fimmu.2021.758368)
Supplement: Supplementary file 1 [file DataSheet_1.pdf]

frontiers

Supplementary Material

Supplementary Table 1. Scoring index of the clinical signs

Signs	Score
Temperature	0-5
Inappetence	0-6
Recumbency	0-6
Skin discoloration	0-3
Respiratory changes	0-6
Nasal discharge	0-2
Eyes/conjunctiva	0-1
Body condition	0-2

Supplementary Table 2. Antibodies used in flow cytometry

Panel	Antibody	Clone	Isotype	Specie	Dilution	Supplier
1	Anti-porcine CD3-PE	BB23-8E6-8C	IgG2a	Mouse	1 : 200	BD Biosciences
	Anti-porcine CD4-PerCP-Cy5.5	74-12-4	IgG2b	Mouse	1 : 100	BD Biosciences
	Anti-porcine CD8β-FITC	PPT23	IgG1	Mouse	1 : 400	Bio-Rad
	Anti-porcine IFN-γ-AF647	P2G10	IgG1	Mouse	1 : 800	BD Biosciences
	Anti-human TNF-α-BV421	Mab11	IgG1	Mouse	1 : 100	BioLegend
	Anti-porcine IL-2	A150D3F1	IgG2a	Mouse	1 : 500	Thermo Fisher Scientific
	Anti-mouse IgG2a-PE-Cy7	m2a-15F8	IgG1	Rat	1 : 400	Thermo Fisher Scientific
2	Anti-porcine CD3-AF647	BB23-8E6-8C	IgG2a	Mouse	1 : 200	BD Biosciences
	Anti-porcine CD4-PerCP-Cy5.5	74-12-4	IgG2b	Mouse	1 : 100	BD Biosciences
	Anti-porcine CD8β-FITC	PPT23	IgG21	Mouse	1 : 400	Bio-Rad
	Anti-human IL-17-PE	eBio64DEC17	IgG1	Mouse	1 : 40	Thermo Fisher Scientific
	Anti-human IL-4-BV421	MP4-25D2	IgG1	Mouse	1 : 40	BioLegend
3	Anti-porcine CD2	MSA4	IgG2a	Mouse	1 : 800	Kingfisher Biotech
	Anti-porcine TCR1 Delta Chain	PGBL22A	IgG1	Mouse	1 : 800	Kingfisher Biotech
	Anti-Mouse IgG2a-PE-Cy7	m2a-15F8	IgG1	Rat	1 : 400	Thermo Fisher Scientific
	Anti-mouse IgG1- PerCP-Cy5.5	RMG1-1	IgG	Rat	1 : 200	BioLegend
	Anti-porcine IFN-γ-PE	P2G10	IgG1	Mouse	1 : 800	BD Biosciences
	Anti-human TNF-α-BV421	Mab11	IgG1	Mouse	1 : 100	BioLegend
	Anti-porcine IL-17-FITC	eBio64DEC17	IgG1	Mouse	1 : 40	Thermo Fisher Scientific

Supplementary Table 3. Body temperature recordings (°C) after the challenge

DPC[a]	Naïve						Ctrl + H3N2						Ctrl + PRRSV-2					
Pig #	1	2	3	4	5	6	7	8	9	10	11	12	13	14	15	16	17	18
-1	39.7	39.1	38.8	39.7	39.5	39.3	39.3	39.5	39.3	39.3	39.4	39.4	39.2	38.8	39.8	39.5	38.8	38.8
0	38.9	39.6	39.2	38.6	39.2	39.0	38.5	38.3	38.7	38.9	39.0	38.8	39.3	39.2	39.6	39.5	38.8	39.6
1	39.7	39.8	39.2	39.5	39.8	39.7	39.6	39.4	38.8	39.1	39.2	39.7	39.3	39.2	39.8	39.3	39.3	39.6
2	39.7	39.5	39.1	39.5	39.8	39.4	39.3	38.9	38.4	39.6	39.2	**40.0**	38.1	39	39.5	39.2	38.9	39.3
3	39.7	39.8	39.4	39.5	39.7	39.7	38.8	38.7	39.0	38.4	38.8	38.6	39.8	39.6	39.3	39	38.8	39.7
4	39.2	39.6	39.1	39.4	39.5	39.4	39.3	39.0	39.3	38.9	38.9	39.0	**40.1**	39.9	**40.0**	39.4	38.9	39.9

DPC[a]	Ctrl + PRRSV-2/H3N2						Vac + PRRSV-2						Vac + PRRSV-2/H3N2					
Pig #	19	20	21	22	23	24	25	26	27	28	29	30	31	32	33	34	35	36
-1	38.0	39.1	38.7	39.2	38.7	38.8	39.1	39.7	39.3	38.8	38.8	38.7	38.3	38.6	39.1	38.9	38.3	38.8
0	38.8	39.2	39.2	38.7	38.9	39.3	38.6	38.3	38.3	38.4	38.4	38.4	38.9	39.2	38.6	39.0	38.6	38.5
1	39.1	39.1	39.3	39.2	38.9	39.0	39.3	39.7	39.6	39.3	39.4	39.3	39.7	39.5	**40.0**	39.1	39.3	39.6
2	38.8	38.6	39.0	39.0	39.0	39.6	39.4	39.7	39.7	39.6	39.9	39.4	**40.3**	**40.1**	39.3	**40.9**	39.3	39.5
3	38.9	39.7	39.5	39.8	39.7	35.9	38.9	39.5	39.5	39.4	39.7	39.0	39.2	39.1	39.5	**40.6**	39.5	39.3
4	38.9	**40.4**	39.4	39.6	38.9	39.5	39.1	39.1	38.4	38.6	38.6	38.2	39.6	39.6	39.3	**40.7**	39.3	39.5

a: days post challenge

Supplementary Table 4. Percentage identity of amino acid sequences between PRRS MLV (GenBank AF066183.4) and PRRSV-2 16CB02 (GenBank MZ700336) strain proteins

Protein	Percentage identity
GP2	92%
GP3	86%
GP4	89%
GP5	87%
M	96%

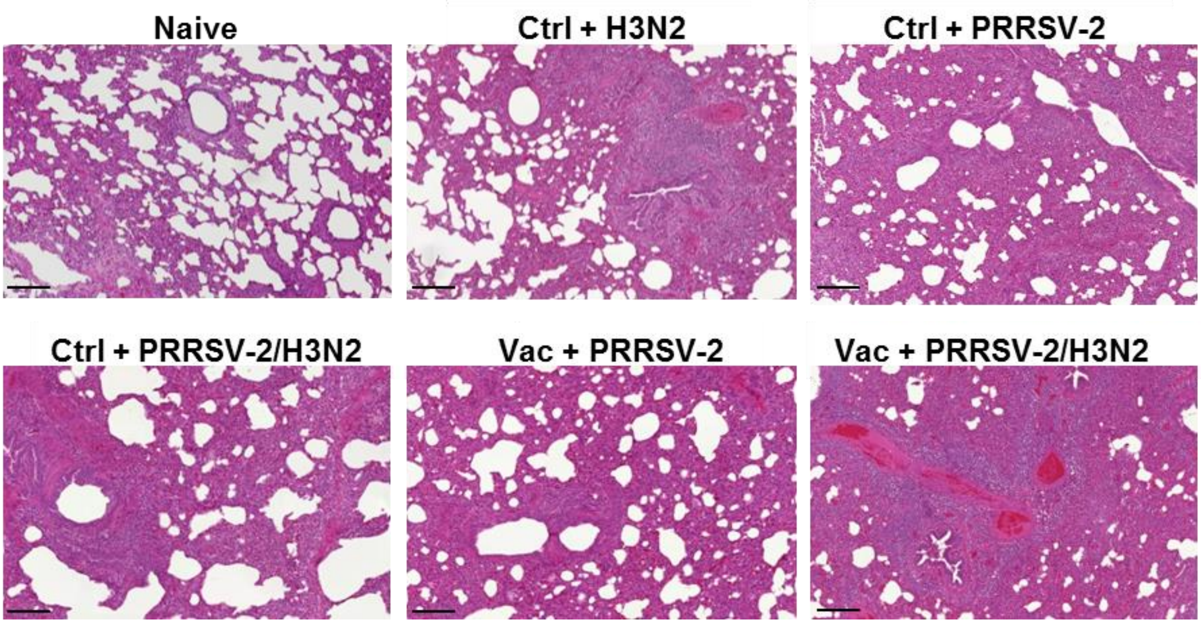

Supplementary Figure 1. Histopathology of lungs. Sections of cranial, cardiac and diaphragmatic lung lobes collected at 5 dpc were stained with H&E and microscopic lesions scored. Representative images of histologic samples from each group (n=6 per group) are shown (original magnification x 100; bar 100 μm).

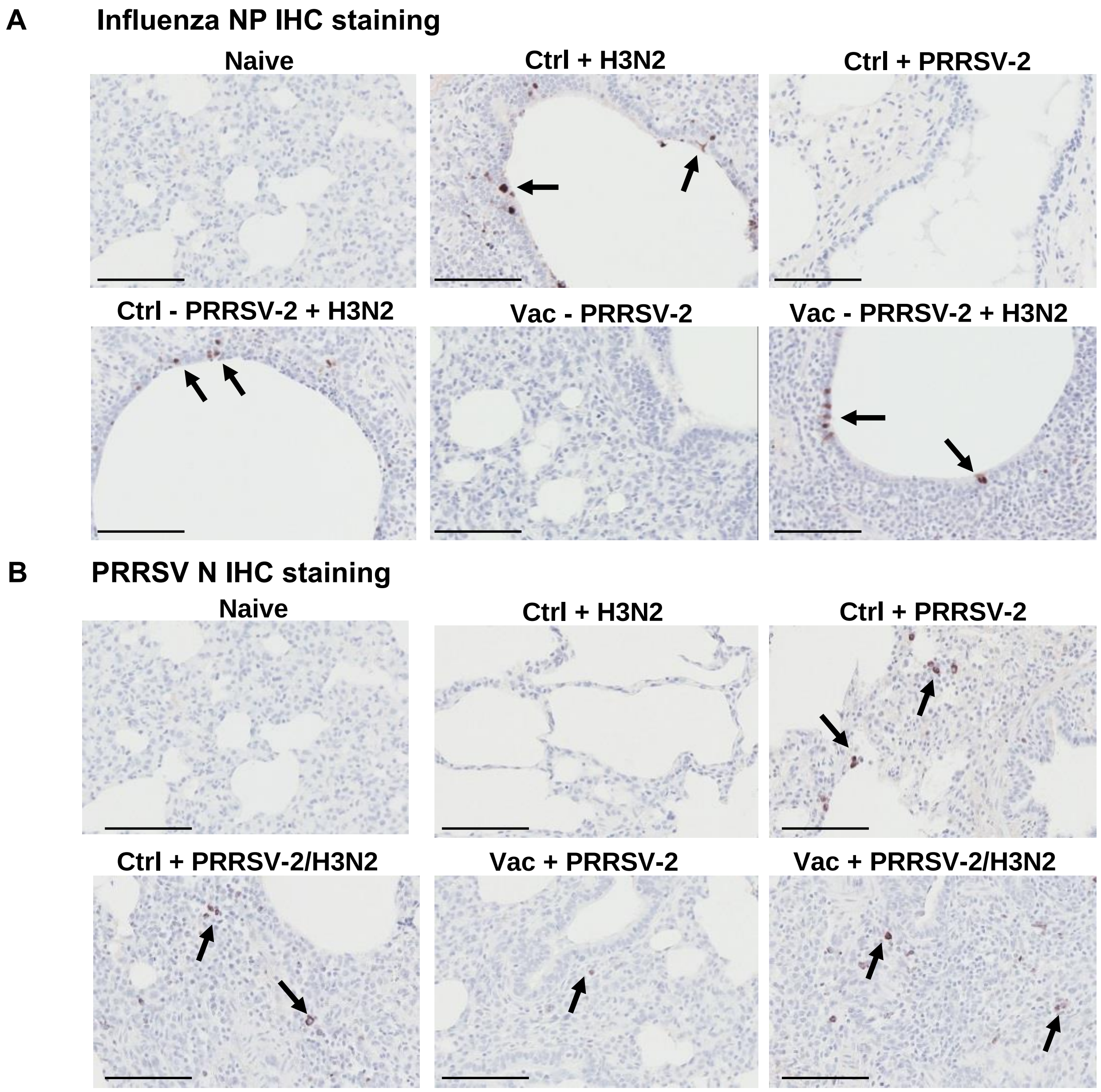

Supplementary Figure 2. Immunohistochemical staining for virus detection. Sections of cranial, cardiac and diaphragmatic lung lobes collected at 5 dpc were stained for virus. **(A)** Lung sections stained using an anti-influenza NP mAb are shown and presence of NP-positive cells in bronchiolar epithelial cells are indicated by the black arrows. **(B)** Lung sections stained with an anti-PRRSV N mAb are shown and N-positive cells are indicated by the black arrows. Immunohistochemical staining of representative lungs for each group (n=6 per group) are shown (original magnification x 400; scale bar 200 μm).

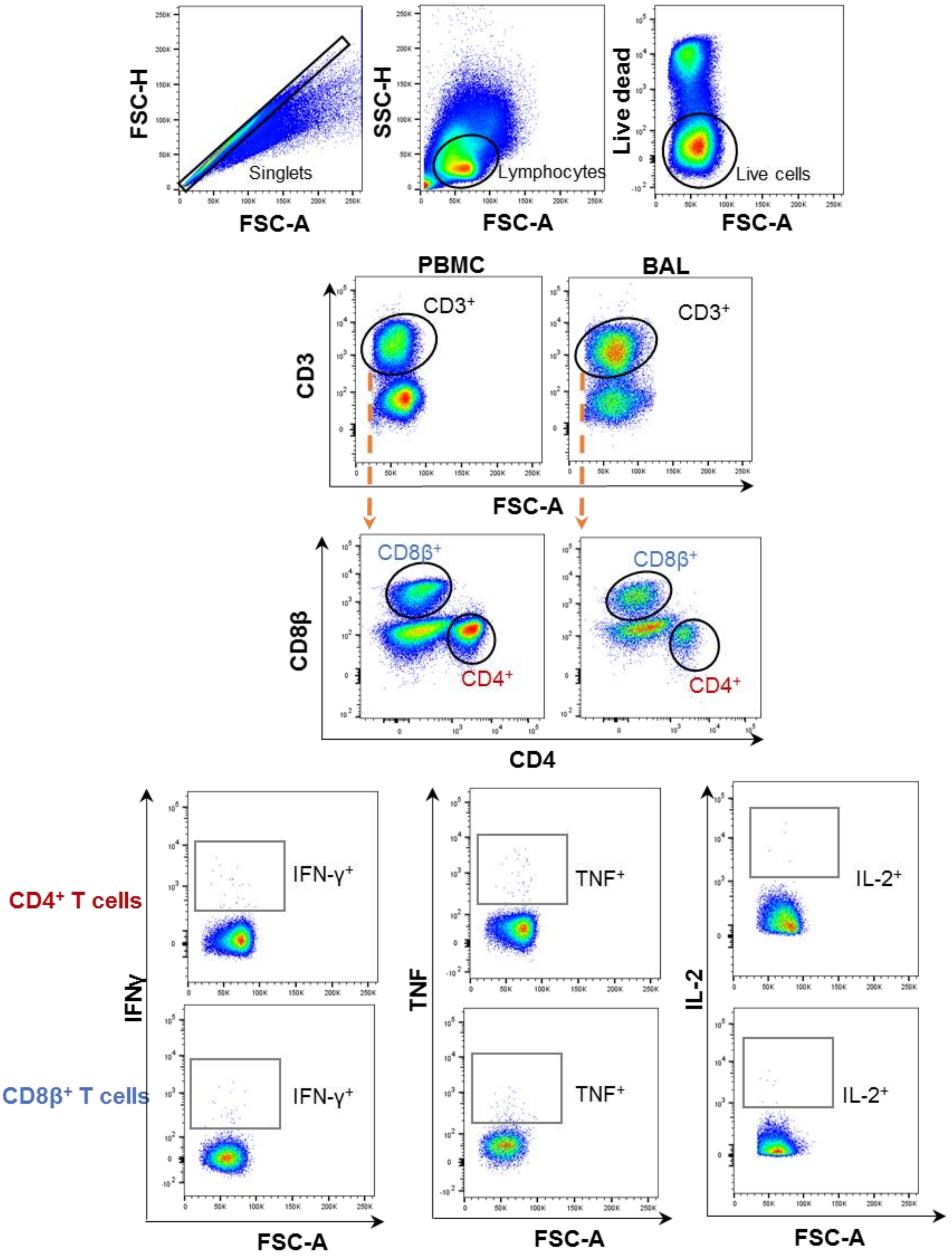

Supplementary Figure 3. Intracellular cytokine staining gating strategy. Successive gates were applied to identify singlet cells, FSC/SSC defined lymphocytes, live, CD3+ and subsequently CD4+ or CD8β+ T cells in the PBMC and BALF cells. Representative plots showing IFN-γ-, TNF- and IL-2- producing cells within CD4+ and CD8β+ T cell gates are indicated in the lower quadrants.

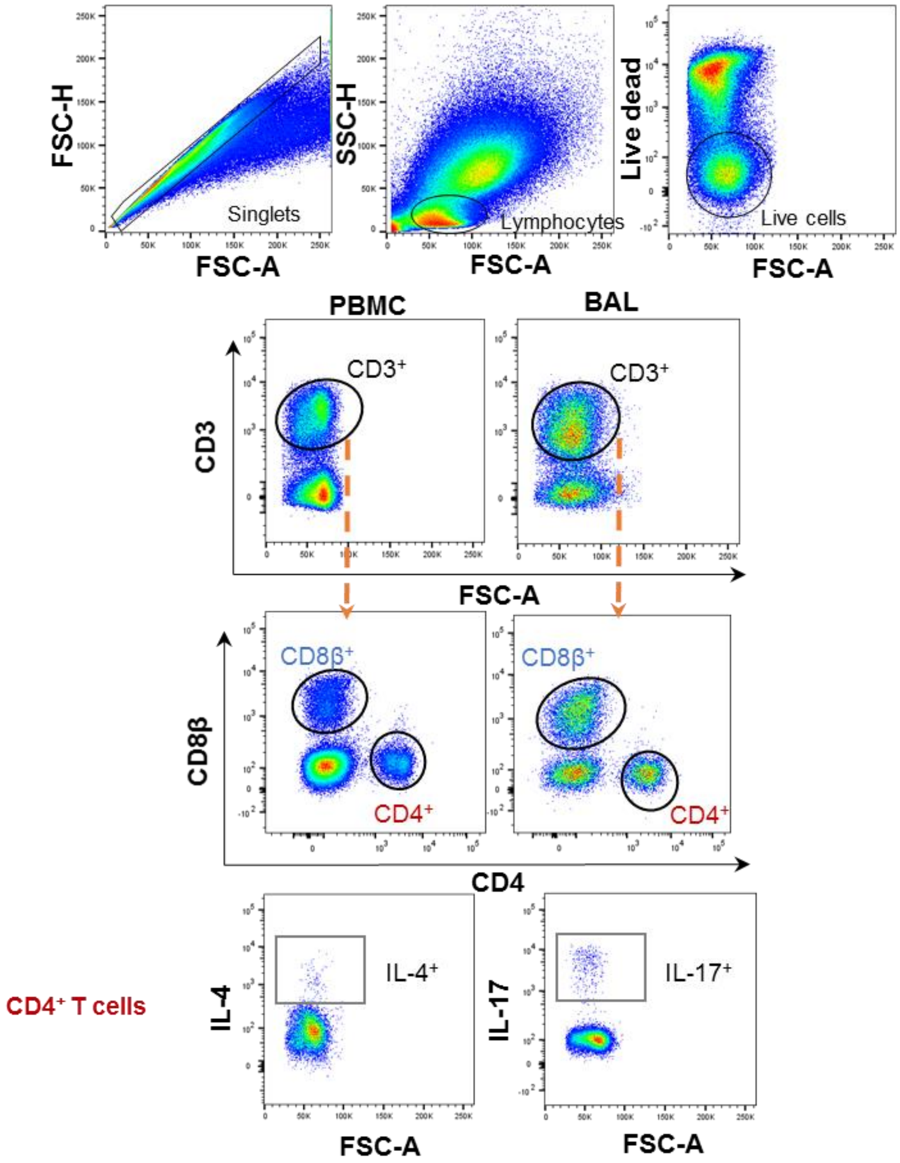

Supplementary Figure 4. Intracellular cytokine staining gating strategy. Successive gates were applied to identify singlet cells, FSC/SSC defined lymphocytes, live, CD3$^+$ and subsequently CD4$^+$ or CD8β$^+$ T cells in the PBMC and BAL cells. IL-4- and IL-17- producing cells within CD4$^+$ T cell gates are indicated in the lower quadrants.

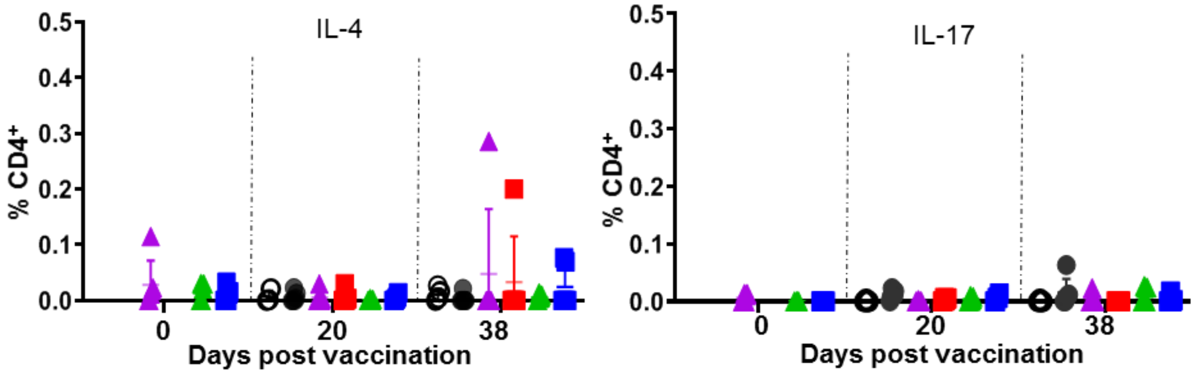

Supplementary Figure 5. T cell responses against PRRSV-2. PBMC cells were restimulated *in vitro* with PRRSV-2 or cultured with medium as previously described in **Figure 4**. Frequency of IL-4- and IL-17- secreting cells within the $CD4^+$ and $CD8\beta^+$ T cells are shown. The corrected frequencies (percentage of cytokine-producing cells subtracted with medium only) of each individual pigs and the mean ± SD are displayed (n=5-6 per group).

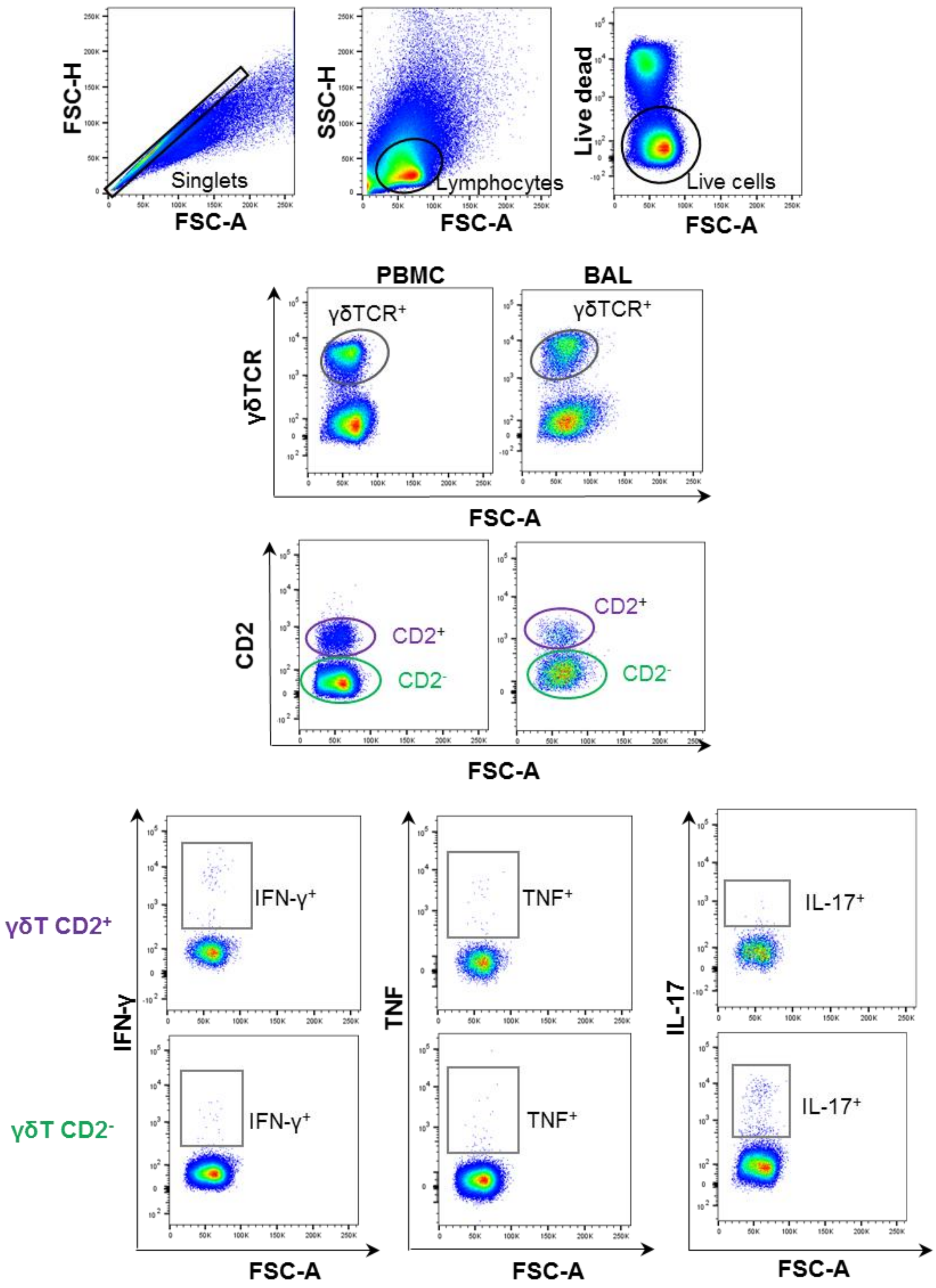

Supplementary Figure 6. Intracellular cytokine staining gating strategy. Successive gates were applied to identify singlet cells, FSC/SSC defined lymphocytes, live, γδTCR^{+} and subsequently CD2^{+} or CD2^{-} cells in the PBMC and BAL cells. IFN-γ-, TNF- and IL-17-producing cells within CD2^{+} and CD2^{-} γδT cell gates are indicated in the lower quadrants.

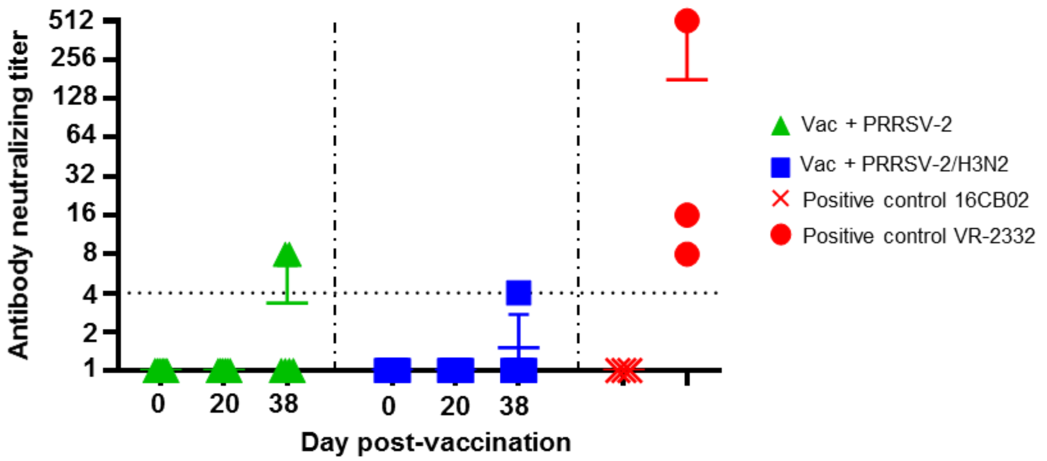

Supplementary Figure 7. Neutralizing Ab titer against PRRSV-2 VR-2332. Neutralizing Ab titer against PRRSV-2 VR-2332 in the serum of PRRS-vaccinated pigs at 0, 20 and 38 are shown. Positive controls were included (n=3 per group, red symbols). Each pig serum is shown as a symbol within the indicated group (n=6 per group) and the mean ± SD is represented. Starting dilution is indicated with dashed lines.
